# A decade of investments in monitoring the HIV epidemic: how far have we come? A descriptive analysis

**DOI:** 10.1186/1478-4505-12-62

**Published:** 2014-10-16

**Authors:** Tobias Alfven, Lotus McDougal, Luisa Frescura, Christian Aran, Paul Amler, Wayne Gill

**Affiliations:** Strategic Information and Monitoring Division, UNAIDS, 20 Avenue Appia, 1211 Geneva, Switzerland; University of California San Diego, 9500 Gilman Drive 0507, La Jolla, California USA; University of Technology Berlin, Straße des 17.Juni 135, Berlin, Germany; Strategic Information, Soul Systems, 13824 22nd Sideroad, Georgetown, ON Canada

**Keywords:** AIDS spending, United Nations General Assembly Special Session on HIV/AIDS (UNGASS), HIV, Monitoring and evaluation, National Composite Policy Index

## Abstract

**Background:**

The 2001 Declaration of Commitment (DoC) adopted by the General Assembly Special Session on HIV/AIDS (UNGASS) included a call to monitor national responses to the HIV epidemic. Since the DoC, efforts and investments have been made globally to strengthen countries’ HIV monitoring and evaluation (M&E) capacity. This analysis aims to quantify HIV M&E investments, commitments, capacity, and performance during the last decade in order to assess the success and challenges of national and global HIV M&E systems.

**Methods:**

M&E spending and performance was assessed using data from UNGASS country progress reports. The National Composite Policy Index (NCPI) was used to measure government commitment, government engagement, partner/civil society engagement, and data generation, as well as to generate a composite HIV M&E System Capacity Index (MESCI) score. Analyses were restricted to low and middle income countries (LMICs) who submitted NCPI reports in 2006, 2008, and 2010 (n =78).

**Results:**

Government commitment to HIV M&E increased considerably between 2006 and 2008 but decreased between 2008 and 2010. The percentage of total AIDS spending allocated to HIV M&E increased from 1.1% to 1.4%, between 2007 and 2010, in high-burden LMICs. Partner/civil society engagement and data generation capacity improved between 2006 and 2010 in the high-burden countries. The HIV MESCI increased from 2006 to 2008 in high-burden countries (78% to 94%), as well as in other LMICs (70% to 77%), and remained relatively stable in 2010 (91% in high-burden countries, 79% in other LMICs). Among high-burden countries, M&E system performance increased from 52% in 2006 to 89% in 2010.

**Conclusions:**

The last decade has seen increased commitments and spending on HIV M&E, as well as improved M&E capacity and more available data on the HIV epidemic in both high-burden and other LMICs. However, challenges remain in the global M&E of the AIDS epidemic as we approach the 2015 Millennium Development Goal targets.

**Electronic supplementary material:**

The online version of this article (doi:10.1186/1478-4505-12-62) contains supplementary material, which is available to authorized users.

## Background

Since the 2001 United Nations General Assembly Special Session (UNGASS) Declaration of Commitment on HIV/AIDS [1], countries and the international community have channeled unprecedented political and financial support to the fight against AIDS. Investments increased eight-fold during the 10 years that followed the Declaration to over $16.8 billion in 2011 and $18.9 billion in 2012 [2]. There has been a parallel increase in focus on accountability to ensure that this funding is spent responsibly and to enable programmatic improvement, policy change, and a greater understanding of the evolving nature of the AIDS epidemic. This is dependent on high quality, representative data, which is in large part gathered from national monitoring and evaluation (M&E) efforts. These data, and the related data systems, are essential to enable countries to have greater ownership of their response by allowing them to plan for, collect, analyze, disseminate, and use HIV-related data to inform the national response [3]. Strengthening national M&E system capacity is therefore a critical component of combating the AIDS epidemic. Country-owned HIV M&E systems without duplication are supported by the “Three Ones” principles [4], which sought to create a nationally unified data system to support the information needs of a dramatically escalating HIV epidemic.

Creating, maintaining, and improving national health information systems requires sustained investment of political, human, and financial resources [5]. General guidance states that 5% to 10% of the national AIDS budget should be used for HIV M&E activities [6]. There has been substantial investment in HIV M&E systems in the last 10 years [7], as well as several efforts to define the basic components of a functional HIV M&E system [3, 8]. However, to our knowledge, there has not been a quantitative assessment of M&E investments and national HIV M&E system capacity on a global scale.

The goal of this paper is to quantify HIV M&E investments, commitments, capacity, and performance in the decade following the Declaration of Commitment to inform the current global discussion on expanding access and use of development data, and ensure increased support for accountability systems in the context of the post-2015 agenda.

## Methods

### Data source

Data on M&E spending, systems, and performance were derived from UNGASS country reports submitted in 2006 [9], 2008 [10], and 2010 [11]. Country Progress Reports, including a core set of indicators, have been submitted to the Joint United Nations Programme on HIV/AIDS (UNAIDS) every second year since 2004 as part of the UNGASS reporting system [11]. Information on government commitment, government engagement, partner/civil society engagement, data generation, and data dissemination/utilization at the national level was taken from the National Composite Policy Index (NCPI) questionnaire [11], which is an integral part of the UNGASS process and the most routinely collected data globally on M&E system capacity. The NCPI is a two-part questionnaire, the first section of which solicits government feedback on the HIV/AIDS strategic plan, political support, prevention, treatment, care and support, and M&E. The second section is administered to civil society organizations and bilateral and multilateral agencies, and assesses human rights, civil society involvement, prevention and treatment, and care and support. Country response rates over the 2006, 2008, and 2010 reporting cycles ranged from 50% to 90% (Table 1).

**Table 1:** **UNGASS and NCPI reporting response rates, 2006–2010**

	2006		2008		2010		2006–2010 ^1^	
	N	%	N	%	N	%	N	%
**All countries (n =192)**								
Submitted UNGASS report	143	75%	153	80%	182	95%	129	67%
Submitted NCPI report	95	50%	137	71%	172	90%	86	45%
**Low- and middle-income countries (n =145)**								
Submitted UNGASS report	116	81%	125	86%	141	97%	110	76%
Submitted NCPI report	82	57%	117	81%	135	93%	78	54%

^1^Countries who submitted reports in 2006, 2008, and 2010.

### Sample

These analyses were restricted to low- and middle-income countries (LMICs) who submitted NCPI questionnaires in each of the 2006, 2008, and 2010 reporting cycles (n =78) in order to assess trends over time. The 2004 NCPI reporting was excluded as it did not include a M&E section.

The 78 LMICs who submitted NCPI questionnaires in 2006, 2008, and 2010 were subdivided into the 22 LMICs with the highest number of people living with HIV, hereafter known as the “high-burden countries”, compared with 56 other LMICs (hereafter known as “other LMICs”). The high-burden countries are Angola, Botswana, Brazil, Burundi, Cameroon, Côte d’Ivoire, India, Indonesia, Kenya, Lesotho, Malawi, Mozambique, Namibia, Nigeria, Russian Federation, Rwanda, South Africa, Swaziland, Uganda, Ukraine, Vietnam, and Zambia. The other LMICs are Algeria, Argentina, Armenia, Bangladesh, Belarus, Belize, Benin, Bulgaria, Burkina Faso, Cambodia, Chile, Colombia, Comoros, Congo, Costa Rica, Democratic Republic of the Congo, Dominica, Ecuador, El Salvador, Ethiopia, Fiji, Gabon, Georgia, Guatemala, Guinea, Guinea-Bissau, Islamic Republic of Iran, Kyrgyzstan, Lao People’s Democratic Republic, Madagascar, Malaysia, Mali, Marshall Islands, Mauritius, Mongolia, Morocco, Nepal, Nicaragua, Pakistan, Palau, Panama, Paraguay, Philippines, Republic of Moldova, Romania, Saint Kitts and Nevis, Saint Lucia, Saint Vincent and the Grenadines, Senegal, Somalia, Sri Lanka, Tajikistan, the former Yugoslav Republic of Macedonia, Togo, Turkey, and Uruguay.

This analysis focuses on four primary areas: government commitments to HIV M&E systems, HIV M&E expenditures, HIV M&E system capacity, and HIV M&E system performance.

### Government commitments

Government commitment was measured using three questions in the NCPI, outlined in Additional file 1. The scores for each question were averaged to create a domain score for each country, as explained further below and detailed in Additional file 1. There were two questions that could not be included in the domain score as they were not asked in all three reporting cycles, but are displayed in Table 2.

**Table 2:** **Summary of government commitment to HIV monitoring and evaluation systems among low- and middle-income countries who completed the NCPI questionnaires in 2006, 2008, and 2010**

		2006		2008		2010	
		N ^1^	Score	N ^1^	Score	N ^1^	Score
Government commitment							
HIV M&E plan	High-burden LMICs	22	64%	22	95%	22	95%
			(44–83)		(89–100)		(86–100)
	Other LMICs	46	61%	56	83%	55	83%
			(50–72)		(75–91)		(75–91)
HIV M&E plan budget	High-burden LMICs	20	78%	22	98%	21	95%
			(58–97)		(93–100)		(85–100)
	Other LMICs	40	65%	52	75%	39	78%
			(51–79)		(66–84)		(65–91)
Secured funding for HIV M&E plan budget	High-burden LMICs	16	69%	20	70%	20	60%
			(43–94)		(48–92)		(36–84)
	Other LMICs	25	72%	39	69%	28	43%
			(53–91)		(54–84)		(23–62)
HIV M&E expenditure monitoring^2^	High-burden LMICs	–	–	–	–	20	80%
							(61–99)
	Other LMICs	–	–	–	–	25	80%
							(63–97)
HIV M&E framework present in national strategic plan^2^	High-burden LMICs	–	–	22	100%	22	91%
							(78–100)
	Other LMICs	–	–	55	91%	56	93%
					(83–99)		(86–100)
Government commitment score	High-burden LMICs	22	65%	22	88%	22	81%
			(49–81)		(80–96)		(70–92)
	Other LMICs	46	57%	56	72%	55	65%
			(47–68)		(65–80)		(59–72)

Note: Confidence levels above 100% have been capped at 100%.

^1^Indicates the number of countries with a response for that question.

^2^Excluded from calculation of domain score as the question was not asked in all three reporting cycles.

### HIV M&E expenditures

HIV M&E expenditures were drawn from UNGASS Indicator 1 which measures levels of HIV/AIDS spending and is collected through a standard reporting form [11]. This national funding matrix is a two-dimensional matrix showing how funds are spent at the national level and where those funds are sourced. This is accomplished by relating financing sources (funding entities that disburse money to agents) with AIDS spending categories (goods, services, and activities delivered as part of the HIV response).

Domestic spending can be classified in the National Funding Matrix under 89 specific spending categories. The spending categories follow a functional classification described in the National AIDS Spending classifications and definitions [12, 13]. We analyzed the spending trends and funding sources of M&E expenditures which are categorized under AIDS spending category 04.03 “Monitoring and evaluation” [12]. In addition, analyses included a broader sub-set of spending categories which we considered important components of an adequate M&E framework, referred to as “M&E*+*” which includes the following: 04.04 (Operations research), 04.05 (Serological surveillance), 04.06 (HIV drug-resistance surveillance), 04.08 (Information technology), and 04.09 (Patient tracking) in addition to 04.03 “Monitoring and evaluation”.

A descriptive analysis of HIV and health expenditures in the time span from 2007 to 2010 of the subset of 43 LMICs with reported data for at least two of these four years was conducted. Spending data prior to 2007 was not available. Comparable data was used to reveal the trend of the share of M&E and M&E + out of total spending on HIV/AIDS. When necessary, the quantity of data points was broadened by conducting a linear interpolation between two known points in the dataset.

### HIV M&E system capacity

HIV M&E system capacity was calculated as a composite of three domains: government engagement, partner/civil society engagement, and data generation. All NCPI questions related to M&E from both government and civil society sections administered as part of the 2006, 2008, and 2010 UNGASS reporting cycle were reviewed. Questions that remained consistent over the three cycles and were relevant to HIV M&E system capacity were grouped into three domains: government engagement, partner and civil society engagement, and data generation. Percentage scores for each question were calculated using the scoring rubric outlined in Additional file 2.

### Index construction and analysis

The three domains, (i) government engagement, (ii) partner and civil society engagement, and (iii) data generation, were also aggregated to form an index score, the HIV M&E System Capacity Index (MESCI) (see Additional file 2 for individual components and scoring calculations). While information on data utilization was collected through the NCPI, only one question was asked in all three reporting cycles, and is therefore not included in domain or index calculations.

To calculate the MESCI, an arithmetic mean was generated for each of the three domains in the MESCI using the scoring methodology outlined in Additional file 2. Means included only questions that were asked in 2006, 2008, and 2010 NCPI cycles. The three domain scores were then averaged to generate the overall HIV M&E System Capacity Index, which ranges from 0% to 100%, for each country and each time-point. MESCI component, domain, and overall scores, including 95% confidence intervals, are presented for each round of UNGASS reporting, stratified by high-burden countries vs. other LMICs. Countries that did not answer a particular question were given a missing value for that question, rather than imputing a response.

### HIV M&E performance

UNGASS indicator reporting from 2006 to 2010 was used to represent HIV M&E system performance. Reported UNGASS indicators were analyzed to assess levels of country reporting for three data streams, namely indicators measured from nationally representative, population-based surveys (UNGASS indicator numbers 7, 13 15, 16, 17), behavioral surveillance for key populations at higher risk of HIV, i.e., men who have sex with men, sex workers, and people who inject drugs (UNGASS indicator numbers 8, 14, 18, 19, 20, 21, 23), and service delivery metrics were derived from program data (UNGASS indicator numbers 4,5,6,24) (Additional file 3) [9–11]. Countries reporting any of the indicators in each data stream were counted as having reported in that particular data stream. The scores for each of the three streams were averaged to create an HIV M&E performance score. We also examined the number of countries able to report program data on antiretroviral therapy by sex; this metric was not available in 2008 and is therefore not included in the performance score calculations.

All analyses were conducted using SAS v. 9.2.

## Results

### Government commitment to HIV M&E

Levels of government commitment to HIV M&E generally increased between 2006 and 2008 (Table 2). This was particularly true for countries reporting having an HIV M&E plan in place, which significantly increased from 64% to 95% in high-burden countries, and from 61% to 83% in other LMICs. The percentage of countries reporting secured funding for a HIV M&E plan budget was stable between 2006 and 2008, but decreased between 2008 and 2010. The overall government commitment domain score in high-burden countries increased considerably between 2006 and 2008, but decreased slightly from 2008 to 2010 (65% to 88% to 81%, respectively).

### HIV M&E expenditures

Over time, reporting on HIV M&E expenditure increased from 2004 until 2009 when the number of countries reported peaked, since slightly fewer countries reported in 2010. The total AIDS spending for the 43 LMICs studied grew from 2007 to 2010, reaching a total of $3.9 billion (Table 3). In these same countries, the percentage of that money that was spent on both M&E and M&E + grew steadily from 2007 to 2010 (from 1.16% to 1.38%, and from 1.55% to 1.91%, respectively). The percentage of total AIDS spending allotted to M&E and M&E + generally increased over time among high-burden LMICs. However, high-burden LMICs reported a lower percentage of funds going to M&E + than that seen in other LMICs in all years analyzed; the same was true for three of four years of M&E spending (2007, 2008, and 2010).

**Table 3:** **Total AIDS spending and HIV M&E spending as a percent of total AIDS spending for low- and middle-income countries (n =43), 2007–2010**

	2007		2008		2009		2010	
	**Total in $1,000 USD**		**Total in $1,000 USD**		**Total in $1,000 USD**		**Total in $1,000 USD**	
**Total AIDS spending**								
All LMICs	2,671,854		3,447,988		3,486,981		3,905,516	
High-burden LMICs	1,841,206		2,423,261		2,464,152		2,775,669	
Other LMICs	337,539		426,205.42		410,692.46		469,596	
**M&E**	**Total in $1,000 USD**	**%**	**Total in $1,000 USD**	**%**	**Total in $1,000 USD**	**%**	**Total in $1,000 USD**	**%**
All LMICs	31,126	1.16%	45,547	1.32%	45,143	1.29%	53,952	1.38%
High-burden LMICs	19,627	1.07%	31,442	1.30%	35,578	1.44%	38,993	1.40%
Other LMICs	4,285	1.27%	5,935	1.39%	5,389	1.31%	6,953	1.48%
**M&E+**	**Total in $1,000 USD**	**%**	**Total in $1,000 USD**	**%**	**Total in $1,000 USD**	**%**	**Total in $1,000 USD**	**%**
All LMICs	41,413	1.55%	54,409	1.58%	59,811	1.72%	74,738	1.91%
High-burden LMICs	22,051	1.20%	36,168	1.49%	39,052	1.58%	42,889	1.55%
Other LMICs	6,820	2.02%	8,479	1.99%	10,792	2.63%	10,160	2.16%

M&E is AIDS spending category 04.03 “Monitoring and evaluation”.

M&E + is 04.04 (Operations research), 04.05 (Serological surveillance), 04.06 (HIV drug-resistance surveillance), 04.08 (Information technology), and 04.09 (Patient tracking) in addition to 04.03 (Monitoring and evaluation).

Low- and middle-income countries (n =43): Algeria, Armenia, Belarus, Bolivia, Botswana, Brazil, Bulgaria, Burkina Faso, Burundi, Cameroon, Cape Verde, Central African Republic, Chad, Colombia, Congo, Costa Rica, Democratic Republic of the Congo, El Salvador, Fiji, Gabon, Ghana, Haiti, Honduras, Indonesia, Kazakhstan, Kenya, Kyrgyzstan, Lao People Democratic Republic, Lesotho, Mali, Mauritius, Myanmar, Niger, Nigeria, Peru, Philippines, Republic of Moldova, Tajikistan, Thailand, The former Yugoslav Republic of Macedonia, Togo, Ukraine, and Vietnam.

High-burden LMICs included in the NCPI analyses (n = 10): Botswana, Brazil, Burundi, Cameroon, Indonesia, Kenya, Lesotho, Nigeria, Ukraine, Vietnam.

Other LMICs included in the NCPI analyses (n = 18): Algeria, Armenia, Belarus, Bulgaria, Burkina Faso, Colombia, Costa Rica, Democratic Republic of the Congo, El Salvador, Fiji, Gabon, Kyrgyzstan, Mali, Mauritius, Philippines, Tajikistan, the Former Yugoslav Republic of Macedonia, Togo.

### HIV M&E system capacity

#### Government engagement

Overall, there were improvements in government engagement from 2006 to 2008 in both high-burden and other LMICs, though these gains decreased by 2010 in high-burden countries (Table 4). However, government engagement was higher over time in high-burden countries (84% in 2006 and 2010) compared to other LMICs (56% in 2006 to 70% in 2010), though the gap did lessen over time. While the presence of a functional national HIV M&E unit was reported by 98% of all high-burden countries and 76% of other LMICs in 2010, only slightly more than 60% of countries in both groups reported that these groups meet regularly (representing a more than 20% decrease from 2008 in high-burden countries).

**Table 4:** **Summary of government and partner/civil society engagement to HIV M&E systems, data generation, and data dissemination/utilization for HIV M&E systems, and the HIV M&E System Capacity Index among low- and middle-income countries who completed NCPI questionnaires in 2006, 2008, and 2010**

		2006		2008		2010	
		N ^1^	Score	N ^1^	Score	N ^1^	Score
			(95% CI)		(95% CI)		(95% CI)
**Government engagement**							
HIV M&E priorities determined through national systems assessment^2^	High-burden LMICs	–	–	–	–	22	100%
	Other LMICs	–	–	–	–	53	72%
							(59–84)
Functional national HIV M&E unit	High-burden LMICs	20	93%	22	95%	22	98%
			(81–100)		(90–100)		(93–100)
	Other LMICs	42	69%	56	71%	54	76%
			(56–82)		(60–81)		(66–86)
National HIV M&E Committee or Working Group that meets regularly	High-burden LMICs	20	73%	22	82%	22	61%
			(53–92)		(71–93)		(52–71)
	Other LMICs	43	59%	56	63%	56	63%
			(46–73)		(53–74)		(52–73)
HIV M&E human capacity plan at national, subnational, and service delivery levels^2^	High-burden LMICs	–	–	–	–	22	86%
							(74–99)
	Other LMICs	–	–	–	–	53	63%
							(53–73)
HIV M&E trainings at national, subnational, and civil society levels	High-burden LMICs	20	85%	19	88%	21	95%
			(71–99)		(75–100)		(90–100)
	Other LMICs	44	44%	46	61%	41	68%
			(31–57)		(51–71)		(58–79)
Government engagement domain score	High-burden LMICs	21	84%	22	89%	22	84%
			(75–92)		(81–96)		(79–89)
	Other LMICs	46	56%	56	66%	56	70%
			(46–66)		(58–73)		(58–79)
**Partner and civil society engagement**							
HIV M&E plan endorsed by key partners	High-burden LMICs	14	93%	20	100%	21	95%
			(77–100)				(85–100)
	Other LMICs	22	82%	46	91%	39	97%
			(64–99)		(83–100)		(92–100)
Mechanisms to ensure major partners submit M&E data/reports to national M&E unit	High-burden LMICs	19	53%	21	95%	21	90%
			(28–77)		(85–100)		(77–100)
	Other LMICs	34	65%	48	73%	36	83%
			(48–82)		(60–86)		(71–96)
HIV M&E plan developed in consultation with civil society	High-burden LMICs	16	75%	20	95%	21	95%
			(51–99)		(85–100)		(85–100)
	Other LMICs	31	81%	47	91%	39	92%
			(66–95)		(83–100)		(84–100)
Perceived extent of civil society inclusion^2^	High-burden LMICs	–	–	–	–	22	61%
							(53–68)
	Other LMICs	–	–	–	–	53	52%
							(45–58)
HIV M&E requirements of key partners aligned with national M&E plan^2^	High-burden LMICs	–	–	19	70%	21	68%
					(60–81)		(58–79)
	Other LMICs	–	–	46	65%	37	71%
					(56–76)		(63–79)
Partner and civil society engagement domain score	High-burden LMICs	20	66%	21	97%	21	94%
			(46–86)		(92–100)		(88–100)
	Other LMICs	36	70%	53	83%	43	91%
			(56–84)		(75–91)		(84–97)
**Data generation**							
HIV M&E plan includes data collection strategy	High-burden LMICs	17	88%	22	100%	21	100%
			(71–100)				
	Other LMICs	33	91%	52	96%	39	97%
			(81–100)		(91–100)		(92–100)
HIV M&E plan data collection strategy components^2^	High-burden LMICs	–	–	22	98%	21	100%
					(93–100)		
	Other LMICs	–	–	52	95%	37	96%
					(90–100)		(92–100)
HIV M&E plan includes well-defined, standardized indicators	High-burden LMICs	17	94%	22	100%	21	100%
			(82–100)				
	Other LMICs	34	88%	52	92%	39	92%
			(77–100)		(85–100)		(84–100)
HIV M&E plan includes guidelines on tools for data collection	High-burden LMICs	17	82%	22	100%	21	100%
			(62–100)				
	Other LMICs	34	85%	52	88%	39	85%
			(73–98)		(79–97)		(73–96)
HIV M&E plan includes strategy for assessing data quality	High-burden LMICs	16	81%	22	86%	21	95%
			(60–100)		(71–100)		(85–100)
	Other LMICs	31	77%	51	71%	39	59%
			(62–93)		(58–84)		(43–75)
HIV M&E plan includes data analysis strategy^2^	High-burden LMICs	–	–	–	–	21	95%
							(85–100)
	Other LMICs	–	–	–	–	39	77%
							(63–91)
HIV M&E plan includes data dissemination and use strategy	High-burden LMICs	17	82%	22	95%	21	95%
			(62–100)		(86–100)		(85–100)
	Other LMICs	32	88%	51	84%	38	79%
			(75–100)		(74–95)		(65–93)
Functional national health information system (HIS)	High-burden LMICs	20	80%	22	95%	22	95%
			(61–99)		(86–100)		(86–100)
	Other LMICs	45	87%	54	91%	53	85%
			(76–97)		(83–99)		(75–95)
Functional subnational HIS	High-burden LMICs	20	75%	19	100%	22	95%
			(54–96)				(86–100)
	Other LMICs	42	81%	49	80%	46	78%
			(69–93)		(68–91)		(66–91)
HIV program coverage monitoring^2^	High-burden LMICs	–	–	22	94%	22	94%
					(88–100)		(90–99)
	Other LMICs	–	–	56	90%	56	92%
					(85–94)		(86–98)
Data generation domain score	High-burden LMICs	21	81%	22	97%	22	97%
			(67–94)		(94–99)		(94–100)
	Other LMICs	46	86%	56	85%	54	82%
			(79–93)		(79–91)		(75–88)
**Data utilization**							
Extent of data usage (planning and implementation)	High-burden LMICs	–	–	22	65%	–	–
					(58–73)		
	Other LMICs	–	–	56	69%	–	–
					(63–75)		
Extent of data usage (developing/revising national AIDS strategy)	High-burden LMICs	–	–	–	–	22	65%
							(58–73)
	Other LMICs	–	–	–	–	56	69%
							(63–75)
Extent of data usage (resource allocation)	High-burden LMICs	–	–	–	–	22	75%
							(68–81)
	Other LMICs	–	–	–	–	54	76%
							(70–81)
Extent of data usage (program improvement)	High-burden LMICs	–	–	–	–	22	73%
							(64–82)
	Other LMICs	–	–	–	–	52	69%
							(64–74)
Annual HIV M&E report	High-burden LMICs	21	86%	21	90%	22	91%
			(69–100)		(77–100)		(78–100)
	Other LMICs	46	74%	56	77%	54	80%
			(61–87)		(65–88)		(69–91)
Data utilization domain score^**3**^	Top 30 LMICs	–	–	–	–	–	–
	Other LMICs	–	–	–	–	–	–
**HIV M&E System Capacity Index**							
HIV M&E System Capacity Index Score	High-burden LMICs	22	78%	22	94%	22	91%
			(68–88)		(90–97)		(89–94)
	Other LMICs	46	70%	56	77%	56	79%
			(62–78)		(71–83)		(73–84)

Note: Confidence levels above 100% have been capped at 100%.

^1^Indicates the number of countries with a response for that question.

^2^Excluded from calculation of domain score, as question was not asked in all three reporting cycles.

^3^No domain score calculated, as only one question was asked in all three survey rounds.

### Partner and civil society engagement

In high-burden LMICs, there was a significant increase in overall partner and civil society engagement from 2006 (66%) to 2008 and 2010 (97% and 94%, respectively) (Table 4). The percentage of high-burden countries reporting mechanisms to ensure major partners submit M&E data/reports to the national HIV M&E unit nearly doubled, from 53% in 2006 to 90% in 2010. Reporting the existence of an HIV M&E plan developed in consultation with civil society also increased in high-burden countries from 75% in 2006 to 95% in 2010. The perceived extent of civil society inclusion (61% in 2010 for high-burden countries) and the alignment of partners’ HIV M&E requirements with national M&E plans (a key component of the “Three Ones”; 68% in 2010) were both lower relative to other scores in this domain for high-burden countries.

### Data generation

Overall, high-burden countries had very high scores in the data generation domain, with improvements from 2006 (81%) to 2008 (97%) that were maintained into 2010 (Table 4). Other LMICs did not report as many improvements over time, and in fact showed decreases between 2008 and 2010 in the number of countries reporting that their national HIV M&E plan included i) guidelines on tools for data collection (88% to 85%), ii) a strategy for assessing data quality (71% to 59%), iii) a data dissemination and use strategy (84% to 79%) and iv) the presence of a functional national and subnational HIS (91% to 85% and 80% to 78%, respectively).

### Data utilization

While no domain score was calculated for data utilization, the percentage of countries producing annual HIV M&E reports increased steadily from 2006 to 2010 across all LMICs (Table 4).

### HIV M&E system capacity index

**Figure 1:**
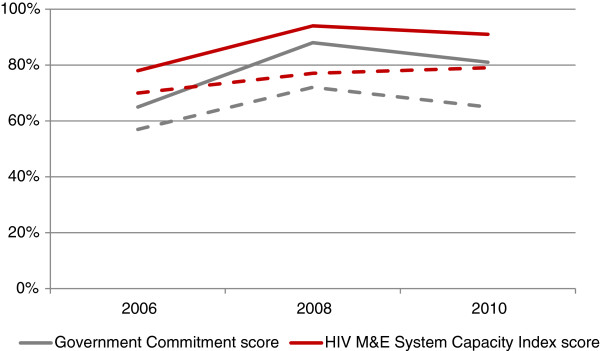
**Government commitment score and HIV Monitoring and Evaluation System Capacity Index Score from 2006 to 2010.** Note: Solid lines represent high-burden LMICs, dotted lines represent other LMICs.

Overall, the index improved between 2006 and 2008, though this improvement was greater in high-burden LMICs (a significant shift from 78% to 94%) than in other LMICs (70% to 77%) (Table 4). High-burden LMICs saw a slight decrease between 2008 and 2010. Among high burden LMICs, the trajectories of government commitment and HIV M&E system capacity map closely onto one another, while in other LMICs, government commitment decreases from 2008–2010 as HIV M&E system capacity increases (Figure 1).

### HIV M&E system performance

M&E system performance generally shows improvements over time among all LMICs regardless of HIV burden (Table 5). For high-burden countries, the overall progress summary score significantly increased from 52% in 2006 to 89% in 2010 and from 37% to 89% for other LMICs. In 2006, only half of the high-burden countries and just one in five of the other LMICs could report on indicators related to the population based indicators. By 2010, this had significantly increased to 95% of high-burden and 84% of other LMICs. The same trend is also seen for indicators related to key populations, with increases from 23% to 73% for high-burden countries and from 50% to 82% for other LMICs, as well as for the service delivery indicators (from 82% to 100% for high-burden and 41% to 100% for other LMICs).

**Figure 2:**
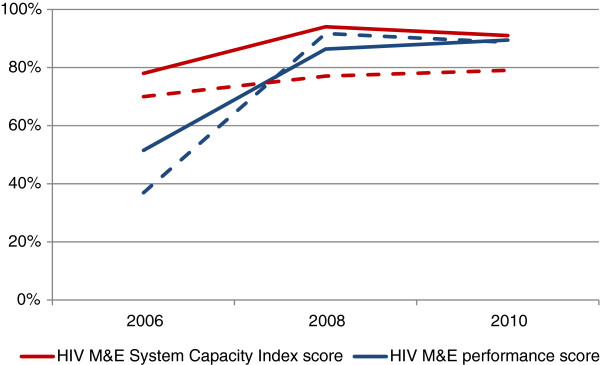
**HIV Monitoring and Evaluation System Capacity Index Score and HIV monitoring and evaluation performance score from 2006 to 2010.** Note: Solid lines represent high-burden LMICs, dotted lines represent other LMICs.

Among high burden countries, both MESCI and HIV M&E performance increase from 2006–2008, and level or slightly decrease by 2010 (Figure 2). In contrast, among other LMICs, HIV M&E performance increases much faster than the MESCI between 2006–2008 (Figure 2).

**Table 5:** **Monitoring and evaluation system performance 2006–2010, as measured through country-reported UNGASS data**

		2006		2008		2010	
		N ^1^	% ^2^	N ^1^	% ^2^	N ^1^	% ^2^
			(95% CI)		(95% CI)		(95% CI)
HIV M&E system performance							
Population-based indicator stream	High-burden LMICs	11	50%	20	91%	21	95%
			(29–71)		(79–100)		(87–100)
	Other LMICs	11	20%	53	95%	47	84%
			(3–36)		(85–100)		(69–99)
Key population indicator stream	High-burden LMICs	5	23%	15	68%	16	73%
			(5–40)		(49–88)		(54–91)
	Other LMICs	28	50%	45	80%	46	82%
			(29–71)		(64–97)		(66–98)
Service delivery indicator stream	High-burden LMICs	18	82%	22	100%	22	100%
			(66–98)				
	Other LMICs	23	41%	56	100%	56	100%
			(21–62)				
Sex disaggregated data on antiretroviral therapy^3^	High-burden LMICs	18	82%	–	–	19	86%
			(66–98)				(72–100)
	Other LMICs	35	63%	–	–	44	79%
			(42–83)				(61–96)
HIV M&E performance score	High-burden LMICs	11	52%	19	86%	20	89%
			(34–69)		(73–100)		(77–100)
	Other LMICs	21	37%	51	92%	50	89%
			(17–57)		(81–100)		(75–100)

Note: Confidence levels above 100% have been capped at 100%.

^1^Indicates the number of countries with a response for that group of questions.

^2^Percent of 22 high-burden LMICs/56 other LMICs submitting any data for each indicator stream.

^3^Excluded from calculation of domain score as the question was not asked in all three reporting cycles.

## Discussion

The rapid expansion of available resources to fight the global AIDS epidemic since the new millennium has brought with it commensurate expectations for accountability at subnational, national, and international levels. Our analysis shows that government commitment to HIV M&E, particularly among high-burden LMICS, has increased considerably since 2006, as has the percentage of AIDS spending allocated to HIV M&E. As government commitments and spending have increased, so have government and partner engagement, data use, and data generation. Over this same time period, HIV M&E system performance has increased by over 70% in high-burden LMICs, and by 140% in other LMICs.

Reported HIV M&E expenditures were low across the board when judged against the 5% to 10% of total AIDS spending as a recommended benchmark [6], never being higher than 1.4% in high-burden countries. While it is likely that there were additional sources of funding for HIV M&E systems beyond those reported in UNGASS reporting, it seems unlikely that they would reach recommended levels. However, the suggested level may also need to be reconsidered at this time as evidenced by the improved performance of the M&E systems at current funding levels.

Many countries still lack the systems to actually track spending on M&E, a barrier that is not confined to the AIDS response. Recognizing this challenge, overall support to national statistical services from international agencies and donor countries has grown considerably, from $US1.0 billion in 2006 to $US2.3 billion in 2010–2012 [14].

The relationship seen between the MESCI score and HIV M&E systems performance (Figure 2) indicates that among high-burden LMICs, countries with high capacity should be better able to produce HIV M&E data to meet national and international needs. This relationship does not seem to hold true for other LMICs, where system performance is higher than system capacity. One potential explanation is that countries with lower burdens of HIV are able to monitor their epidemic with a lower system capacity than that required for high-burden countries.

The mandate for national and international HIV M&E provided in the 2011 Political Declaration on HIV/AIDS [15], to further strengthen and support HIV data systems while working to integrate them with broader national health systems, is a significant shift from the previous strategy of often parallel systems. There has also been a call to use HIV resources more strategically, to encourage greater efficiencies and higher performance [16]. The decrease seen in the number of high-burden countries reporting a National HIV M&E Committee or Working Group that meets regularly may be of concern, as that is one of the main bodies that will be able to guide the integration agenda that has been set for HIV M&E. However, it may also be the evolution of M&E systems from the early and intensive development stages to the current and more established systems maintenance stage. Folding the resources and experience from the HIV response in with those of other health sectors offers the opportunity for synergies that could be used to leverage the maximum benefit out of all systems and resources. Neither the NCPI M&E section nor the MESCI as they are currently structured are able to measure integration between HIV M&E systems and other national M&E systems, but this is an important modification that should be considered in future years.

Since the beginning of the AIDS epidemic, civil society has played a key role in the response in countries all over the world. The engagement in the monitoring of the AIDS epidemic has also been shown to be important [17]. Civil society engagement in the development of M&E plans was reported to be high already in 2008 (around 80% of countries) and increased to more than 90% of countries in 2010. However, the perceived extent of civil society inclusion was lower, at only 60% in high-burden countries and 50% in other LMICs, leaving room for considerable improvements.

This study has several key limitations. Data on government commitments and system capacity is derived from the NCPI, which is self-reported data representing both national government representative’s opinions of the status and performance of their HIV M&E systems and civil society/bilateral/multilateral opinion on perceived inclusion in HIV M&E activities. The nature of response to some of these questions is therefore dependent to some extent on the respondent and their personal measures of performance. Calculations of domain scores and the MESCI were limited to questions that appeared in all three reporting cycles analyzed – as NCPI reporting continues, the contents of each domain may be enhanced by a greater breadth of questions asked over time. Indicator performance and patterns of reporting over time was used as a proxy for M&E system performance in countries. Although there are limitations in this approach, consistent increases in reported data, from varying sources and data collection systems (program data, population-based surveys, behavioral surveillance), indicate that there has been progress in strengthening national M&E systems in recent years.

The most important reason for conducting M&E is to provide the data needed for guiding policy and program implementation. A functional M&E system collates and presents the data in a way that facilitates data use at all levels, including the general public and beneficiaries of HIV services [3]. Countries rated their data utilization for the development/revising of national AIDS strategies, resource allocation and program improvement in 2010 above average in most countries, though there is room for improvement. Accurate and regular data utilization is critical to ensuring epidemiologically appropriate and evidence-based resource allocation; gaps in this process may be in part responsible for the variable resource allocation strategies currently seen across countries [18].

Through the UNGASS reporting mechanism, countries were asked to submit nationally representative data. However, for an effective response it is not sufficient to collect data at the national level but data is also needed at the subnational level, e.g., to identify geographic areas where localized HIV epidemics or specific populations most affected by the epidemic are not being reached by services. In recent years, more focus has been put on these efforts [19].

The gathering of reliable data on the AIDS epidemic and the response to it is of increasing importance for donors such as the Global Fund to Fight AIDS, Tuberculosis, and Malaria, for whom performance-based funding is a core principle [20]. M&E is a cornerstone of this approach and allows donors to make funding decisions against proven measurable results. More recently, the Global Fund to Fight AIDS, Tuberculosis, and Malaria’s New Funding Model has put greater emphasis on impact measurement and allocative efficiency [21]. Further examples include the Investment Framework at a national level [16] and the PEPFAR reporting requirements at a donor level [22].

## Conclusions

This analysis demonstrates increases in all domains of HIV M&E systems assessed between 2006 and 2010, as well as in HIV M&E funding, though the latter remained well below the recommended benchmarks. In this same window, HIV M&E system performance increased substantially. Building national M&E systems requires sustained efforts over long periods of time with effective leadership and coordination [7]. Ongoing investments into effective HIV M&E systems and data utilization are required. However, with the increased resource constraints in many countries, these investments must be spent strategically and according to the specific needs of each country [16]. There is no single M&E system that fits all countries; the systems should be based on an efficient and effective mix of standardized methods of data collection and analyses that meet country and international needs [23]. The 2011 Political Declaration on HIV/AIDS calls for the integration of HIV M&E systems into broader health information systems, a major repositioning for many countries who have invested heavily in parallel M&E systems [4]. This alignment of vertical interventions with the broader health system has gained momentum in light of the health-related Millennium Development Goal targets for which effective scale-up of services for individual diseases relies on the strength of the health system as a whole. In June 2013, a report published by the High-Level Panel on the Post 2015 Development Agenda called for a “New Data Revolution” to strengthen existing data collection systems in order to provide timely data for decision making, monitor delivery gaps, and ensure greater accountability [6]. The mandate to integrate HIV M&E systems within broader health information systems provides new opportunities to leverage and strengthen existing infrastructures. Analyses such as this offer key insights into this process, and should be a routine component of M&E system evaluation.

## Authors’ information

TA, LF, and CA are employees of UNAIDS and were involved in the collection of data used in this study. WG is a former employee of UNAIDS, and was also involved in the collection of data used in this study.

## Electronic supplementary material

Additional file 1: **Government commitment components drawn from the National Composite Policy Index (NCPI).** Summary of individual components and scoring used in the calculation of the HIV M&E System Capacity Index. (DOCX 16 KB)

Additional file 2: **HIV M&E System Capacity Index components drawn from the National Composite Policy Index (NCPI).** Summary of individual components and scoring used in the calculation of the HIV M&E System Capacity Index. (DOCX 45 KB)

Additional file 3: **UNGASS indicators used to assess M&E performance.** Summary of the UNGASS indicators used to assess M&E performance. (DOCX 45 KB)

## Abbreviations

LMICs: Low- and middle-income countries
M&E: Monitoring and evaluation
MESCI: HIV M&E System Capacity Index
NCPI: National Composite policy Index
UNAIDS: United Nations Programme on HIV/AIDS
UNGASS: United Nations General Assembly Special Session.
