# Supplementary material for: A decade of investments in monitoring the HIV epidemic: how far have we come? A descriptive analysis
Source: Health Res Policy Syst. 2014 Oct 16;12:62. doi: 10.1186/1478-4505-12-62 (PMC4210584; doi:10.1186/1478-4505-12-62)
Supplement: Supplementary file 1 — Additional file 1: Government commitment components drawn from the National Composite Policy Index (NCPI). Summary of individual components and scoring used in the calculation of the HIV M&E System Capacity Index. (DOCX 16 KB) [file 12961_2014_357_MOESM1_ESM.docx]

A Decade of Investments in Monitoring the HIV Epidemic:

How far have we come? A descriptive analysis.

Alfven T, McDougal L, Frescura L, Aran C, Amler P, Gill W.

Additional File 1. Government commitment to HIV M&E system components drawn from the National Composite Policy Index (NCPI)

| Indicator | Question | Scoring | NCPI rounds | | |
| --- | --- | --- | --- | --- | --- |
|  |  |  | 2006 | 2008 | 2010 |
| **Government Commitment** | | | | | |
| HIV M&E plan | Does the country have one national Monitoring and Evaluation (M&E) plan? ^1^ | 0=no; 0.5=in progress; 1=yes | ✓ | ✓ | ✓ |
| HIV M&E plan budget | Is there a budget for implementation of the M&E plan? ^1^ | 0=no; 0.5=in progress; 1=yes | ✓ | ✓ | ✓ |
| Secured funding for HIV M&E plan budget | IF YES *[to there being a budget for implementation of the M&E plan]*, has full funding been secured? ^1^ | 0=no; 1=yes | ✓ | ✓ | ✓ |
| HIV M&E expenditure monitoring^2^ | IF YES *[to there being a budget for implementation of the M&E plan]*, are M&E expenditures being monitored? ^1^ | 0=no; 1=yes |  |  | ✓ |
| HIV M&E framework present in national strategic plan^2^ | Does the multisectoral strategy or operational plan include a Monitoring and Evaluation framework? ^1^ | 0=no; 1=yes |  | ✓ | ✓ |
| Government Commitment domain score |  | Arithmetic mean of all domain components | ✓ | ✓ | ✓ |

^1^ Administered to government officials.

^2^ Excluded from calculation of domain score, as question was not asked in all three survey rounds.
