# Supplementary material for: A decade of investments in monitoring the HIV epidemic: how far have we come? A descriptive analysis
Source: Health Res Policy Syst. 2014 Oct 16;12:62. doi: 10.1186/1478-4505-12-62 (PMC4210584; doi:10.1186/1478-4505-12-62)
Supplement: Supplementary file 2 — Additional file 2: HIV M&E System Capacity Index components drawn from the National Composite Policy Index (NCPI). Summary of individual components and scoring used in the calculation of the HIV M&E System Capacity Index. (DOCX 45 KB) [file 12961_2014_357_MOESM2_ESM.docx]

A Decade of Investments in Monitoring the HIV Epidemic:

How far have we come? A descriptive analysis.

Alfven T, McDougal L, Frescura L, Aran C, Amler P, Gill W.

Additional File 2. HIV M&E System Capacity Index components drawn from the National Composite Policy Index (NCPI)

| Indicator | Question | HIV MESCI scoring | NCPI rounds | | |
| --- | --- | --- | --- | --- | --- |
|  |  |  | 2006 | 2008 | 2010 |
| **Government Engagement** | | | | | |
| HIV M&E priorities determined through national systems assessment^1^ | Are M&E priorities determined through a national M&E system assessment? ^2^ | 0=no; 1=yes |  |  | ✓ |
| Functional national HIV M&E unit | Is there a functional national M&E Unit? ^2^ | 0=no; 0.5=in progress; 1=yes | ✓ | ✓ | ✓ |
| National HIV M&E Committee or Working Group that meets regularly | Is there a national M&E Committee or Working Group that meets regularly to coordinate M&E activities? ^2^ | 0=no; 0.5=yes, but meets irregularly; 1=yes, meets regularly | ✓ | ✓ | ✓ |
| HIV M&E human capacity plan at national, subnational and service delivery levels^1^ | Is there a plan for increasing human capacity in M&E at national, subnational and service-delivery levels? ^2^ | 0=no; 0.5=yes, but only addressing some levels; 1=yes, at all levels |  |  | ✓ |
| HIV M&E trainings at national, subnational and civil society levels | In the last year, was training in M&E conducted: ^2^  At national level?  At sub-national level?  At service delivery level including civil  society? | 0=no; 0.33=1 of 3 levels; 0.67=2 of 3 levels; 1=all levels | ✓ | ✓ | ✓ |
| Government Engagement domain score |  | Arithmetic mean of all domain components | ✓ | ✓ | ✓ |
| **Partner and Civil Society Engagement** | | | | | |
| HIV M&E plan endorsed by key partners | If YES *[to country having one national Monitoring and Evaluation (M&E) plan]*, was the M&E plan endorsed by key partners in M&E? ^2^ | 0=no; 1=yes | ✓ | ✓ | ✓ |
| Mechanisms to ensure major partners submit M&E data/reports to national M&E unit | If YES *[to there being a functional national M&E unit*], are there mechanisms in place to ensure that all major implementing partners submit their M&E data/reports to the M&E Unit for inclusion in the national M&E system? ^2^ | 0=no; 1=yes | ✓ | ✓ | ✓ |
| HIV M&E plan developed in consultation with civil society | If YES *[to country having one national Monitoring and Evaluation (M&E) plan],* was the M&E plan developed in consultation with civil society, including people living with HIV? ^2^ | 0=no; 1=yes | ✓ | ✓ | ✓ |
| Perceived extent of civil society inclusion^1^ | To what extent is civil society included in the monitoring and evaluation (M&E) of the HIV response? ^3^  a. Developing the national M&E plan?  b. Participating in the national M&E  committee/working group responsible  for coordination of M&E activities?  c. M&E efforts at local level?  *(0-5 rating for each question)* | cumulative score/15; |  |  | ✓ |
| HIV M&E requirements of key partners aligned with national M&E plan^1^ | If YES *[to country having one national Monitoring and Evaluation (M&E) plan],* have key partners aligned and harmonized their M&E requirements (including indicators) with the national M&E plan? ^2^ | 0=no; 0.33=some; 0.67=most; 1=all |  | ✓ | ✓ |
| Partner and Civil Society Engagement domain score |  | Arithmetic mean of all domain components | ✓ | ✓ | ✓ |
| **Data Generation** | | | | | |
| HIV M&E plan includes data collection strategy | Does the national Monitoring and Evaluation plan include a data collection strategy? ^2^  *[2005/2007 wording: Does the national Monitoring and Evaluation plan include a data collection and analysis strategy?]* | 0=no; 1=yes | ✓ | ✓ | ✓ |
| HIV M&E plan data collection strategy components^1^ | IF YES *[to having a national Monitoring and Evaluation plan],* does it address: ^2^  Routine programme monitoring? (2010 only)  Behavioural surveys? *(2007 wording: Behavioural surveillance)*  HIV surveillance (2007/2010)  Evaluation/research studies (2010 only) | 0=no; 0.25=1 component; 0.5=2 components; 0.75=3 components; 1=all 4 components; 2006 has no components, 2008 has 2 components, 2010 has 4 components |  | ✓ | ✓ |
| HIV M&E plan includes well-defined, standardized indicators | IF *YES [to having a national Monitoring and Evaluation plan],* does it include a well-defined standardized set of indicators? ^2^ | 0=no; 1=yes | ✓ | ✓ | ✓ |
| HIV M&E plan includes guidelines on tools for data collection | IF YES *[to having a national Monitoring and Evaluation plan],* does it include guidelines on tools for data collection? ^2^ | 0=no; 1=yes | ✓ | ✓ | ✓ |
| HIV M&E plan includes strategy for assessing data quality | IF YES *[to having a national Monitoring and Evaluation plan*], does it include a strategy for assessing data quality (i.e., validity, reliability)? ^2^  *[2005:2007 wording: : Does the national Monitoring and Evaluation plan include a strategy for assessing quality and accuracy of data?]* | 0=no; 1=yes | ✓ | ✓ | ✓ |
| HIV M&E plan includes data analysis strategy^1^ | IF YES *[to having a national Monitoring and Evaluation plan]*, does it include a data analysis strategy? ^2^ | 0=no; 1=yes; 2010 only |  |  | ✓ |
| HIV M&E plan includes data dissemination and use strategy | IF YES *[to having a national Monitoring and Evaluation plan]*, does it include a data dissemination and use strategy? ^2^ | 0=no; 1=yes | ✓ | ✓ | ✓ |
| Functional national HIS | Is there a functional Health Information System (HIS)? (*functional means regularly reporting data from health facilities which are aggregated at district level and sent to national level; and data are analysed and used at different levels) at the national level? ^2^ | 0=no; 1=yes | ✓ | ✓ | ✓ |
| Functional subnational HIS | Is there a functional Health Information System (HIS)? (*functional means regularly reporting data from health facilities which are aggregated at district level and sent to national level; and data are analysed and used at different levels) at the subnational level? ^2^ | 0=no; 1=yes | ✓ | ✓ | ✓ |
| HIV programme coverage monitoring^1^ | Is HIV programme coverage being monitored? ^2^  IF YES, is coverage monitored by sex (male, female)?  IF YES, is coverage monitored by population groups?  IF YES, is coverage monitored by geographical area? | 0=no; 0.25=1 component; 0.50=2 components; 0.75=3 components; 1=all 4 components |  | ✓ | ✓ |
| Data generation domain score |  | Arithmetic mean of all domain components | ✓ | ✓ | ✓ |
| **Data Utilization^4^** | | | | | |
| Extent of data usage (planning and implementation | To what extent are M&E data used in planning and implementation? *(0-5 rating)* ^2^ | score/5; 2008 only |  | ✓ |  |
| Extent of data usage (developing/revising national AIDS strategy) | To what extent are M&E data used in developing/revising the national AIDS strategy?*(0-5 rating)* ^2^ | score/5; 2010 only |  |  | ✓ |
| Extent of data usage (resource allocation) | To what extent are M&E data used for resource allocation? *(0-5 rating)* ^2^ | score/5; 2010 only |  |  | ✓ |
| Extent of data usage (programme improvement) | To what extent are M&E data used for programme improvement? *(0-5 rating)* ^2^ | score/5; 2010 only |  |  | ✓ |
| Annual HIV M&E report | Does the country publish at least once a year an M&E report on HIV, including HIV surveillance data? ^2^ | 0=no; 1=yes | ✓ | ✓ | ✓ |
| **HIV M&E System Capacity Index** | | | | | |
| **HIV M&E System Capacity Index** Score |  | Arithmetic mean of all domains | ✓ | ✓ | ✓ |

^1^ Excluded from calculation of domain score, as question was not asked in all three survey rounds.

^2^ Administered to government officials.

^3^ Administered to government officials, civil society organizations, bilateral agencies and UN organizations.

^4^ No domain score was calculated, as only one question was asked in all three survey rounds.
